# Hearing and language development in children with brainstem implants: a systematic review

**DOI:** 10.1016/j.bjorl.2022.07.004

**Published:** 2022-08-05

**Authors:** Quemile Pribs Martins, Bruna de Franceschi Schirmer Gindri, Cristiane Dellinghausen Valim, Laís Ferreira, Fernanda Soares Aurélio Patatt

**Affiliations:** a Universidade Federal de Santa Maria (UFSM), Programa de Pós-Graduação em Distúrbios da Comunicação Humana, Santa Maria, RS, Brazil; b Universidade Federal de Santa Maria (UFSM), Curso de Fonoaudiologia, Santa Maria, RS, Brazil

**Keywords:** Children, Auditory brainstem implant, Hearing, Language

## Abstract

**Objective:**

To present scientific evidence, based on a systematic review of the literature, on the benefit of brainstem implants in auditory rehabilitation and language development in children.

**Methods:**

A systematic search was used to identify studies that contain information about the benefit of brainstem implants in the auditory rehabilitation and language development of children. The review was conducted based on a structured literature search, following the Preferred Reporting Items for Systematic Reviews and Meta-analysis (PRISMA) checklist. The search was carried out in the PubMed, Web of Science and Scopus databases, using the combination “Auditory brainstem implants” AND “Pediatric”, without restriction of language, period, and location. The quality assessment of the articles was performed using the Study Quality Assessment Tools.

**Results:**

Regarding hearing, children with brainstem implants showed sound detection, access to most speech sounds, basic auditory perception skills, recognition of ambient sounds, recognition of some frequently used words and phrases, in addition to some closed-set word discrimination capability. Expressive and comprehensive language were identified in children using auditory brainstem implants, increasing significantly in the short and long terms in most cases; however, in some of the children, such skills remained stable.

**Conclusion:**

The auditory brainstem implant can be considered an effective alternative for children with cochlear malformation and/or auditory nerve deficiency and for those who cannot benefit from cochlear implant surgery.

## Highlights

• Language development is progressive.
• All children acquired basic auditory perception skills.
• Evolution of word identification up to sentence recognition level.
• Alternative to children born with cochlear malformation or auditory nerve deficiency.

## Introduction

The auditory brainstem implant (ABI) is a surgically implanted prosthetic device that directly stimulates the cochlear nucleus complex, located in the brainstem. It is indicated in cases of severe or profound bilateral sensorineural hearing loss, when a cochlear implant (CI) is not feasible, such as in cases of cochlear and/or auditory nerve malformations, or in cases of tumors affecting this nerve. The benefits of ABI are not well described in the scientific literature; however, clinically, this device may become the only alternative for these cases.^1^, ^2^

The development of neural new connections is crucial since birth and is closely related to the central nervous system maturation. During childhood, the neuroplasticity is more susceptible and sensitive to language acquisition, so the integrity of central and peripheral auditory pathways is vital, as they play a significant role in the development of auditory and speech skills in the first years of life.^2^, ^3^, ^4^ Patients with ABI may benefit from increased attention to ambient sounds and speech, promoting improvements in communication and quality of life for these children.^3^, ^4^

The clinical use of ABI was approved in the 2000s and, although there has been a worldwide increase in its use in recent years, there is no consensus in the literature on its clinical applicability and its benefits on auditory skills and language development.^1^, ^2^ Because it is a promising alternative for auditory stimulation in cases of auditory nerve or cochlear malformations, understanding the benefits of ABI is extremely important.

In this context, this systematic review aims to present scientific evidence, based on a systematic literature review, about the benefit of brainstem implants in auditory rehabilitation and language development in children.

## Methods

This systematic review aimed to identify studies that provide information on the benefit of brainstem implants in auditory rehabilitation and language development in children. The review was conducted based on a structured literature search, following the Preferred Reporting Items for Systematic Reviews and Meta-Analyses (PRISMA) checklist.^5^ The methods used in this review were previously published and approved by the International Prospective Register Of Systematic Reviews (PROSPERO) database under number CRD42021264490.

### Eligibility criteria

Eligibility criteria were established based on the PICOS terms answering the following research question: “What is the benefit of ABI in the development of children's auditory and language skills?”. PICOS is an acronym for Target *P*opulation, *I*ntervention, *C*omparison, *O*utcomes, and *S*tudy Types, where the Population (P) of Interest comprises children; Intervention (I) concerns brainstem implants; Comparison (C) does not apply in this study; Outcome (O) refers to the development of listening and language skills; and the admitted Study (S) types were: observational, cohort, cross-sectional, case-control and randomized clinical trials.

The assessed studies were those without language, period and location restrictions that contained information about the evolution or situation of oral language and/or auditory skills after at least six months of ABI and that were evaluated using validated protocols and questionnaires, electrophysiological and/or behavioral tests. In order to be included in the present review, publications should also have a score >80% in the Study Quality Assessment Tools,^6^ showing high-quality study level.

Studies that did not address the desired topic, or that were unclear, or did not answer the research question or that obtained a score <80% in the Study Quality Assessment Tools were excluded.^6^ Non-human studies, narrative reviews, systematic reviews, meta-analyses, non-clinical research (i.e., basic sciences), case reports or series of cases, and abstracts were also excluded.

### Search strategy

Three electronic databases (PubMed, Web of Science and Scopus) were searched between July and August 2021, using individual search strategies by two researchers (QPM e CDV).

The search strategy sought to list keywords that involved brainstem implant and the pediatric population. Therefore, the searches were conducted using the following terms, in addition to the Boolean operator “AND”, thus combined: “Auditory brainstem implants” AND “Pediatric”. There were no restrictions and filters regarding age, year and language of publication.

### Study selection

The articles were independently selected, blindly, by two of the authors, thus preventing any risk of bias. The first selection was performed by reading the title and abstract, according to the established inclusion and exclusion criteria. It is noteworthy that the names of authors and journals were masked to prevent any potential bias and conflicts of interest. Subsequently, the selection was carried out by reading the full-text articles. In cases of discrepancies, a third author (LF) was consulted. Articles that did not comprise the topic of this review or the ones whose full text was not available were excluded.

### Quality analysis

A quality and risk of bias assessment was performed based on the Study Quality Assessment Tools.^6^ The authors independently evaluated each study. The final quality of each article was determined by the following response scores: >80%, good; between 50% and 79%, regular; <50%, bad (Table 1). Only articles with a final score >80%, which are considered to be of high quality, were included.

**Table 1:** Quality assessment of the selected studies.

Year	Authors	Q1	Q2	Q3	Q4	Q5	Q6	Q7	Q8	Q9	Q10	Q11	Q12	Q13	Q14	Quality
2021	Baş B., Yücel E^11^	Y	Y	Y	Y	Y	Y	Y	NA	Y	Y	Y	NA	Y	Y	Good
2020	Fernandes NF, de Queiroz Teles Gomes M, Tsuji RK, Bento RF, Goffi-Gomez MVS^4^	Y	Y	Y	Y	Y	Y	Y	NA	Y	Y	Y	NA	Y	Y	Good
2020	Rajeswaran R, Kameswaran M^10^	Y	Y	Y	Y	Y	Y	Y	NA	Y	Y	Y	NA	Y	Y	Good
2019	Van der Straaten TFK, et al.^15^	Y	Y	Y	Y	Y	Y	Y	NA	Y	Y	Y	NA	Y	Y	Good
2019	Faes J, Gillis S^3^	Y	Y	Y	Y	Y	Y	Y	NA	Y	Y	Y	NA	Y	Y	Good
2020	Aslan, F; Ozkan, H B; Yücel, E∗; Sennaroğlu, G; Bilginer, B; Sennaroğlu, L^16^	Y	Y	Y	Y	Y	Y	Y	NA	Y	Y	Y	NA	Y	Y	Good
2018	Eisenberg LS, et al.^9^	Y	Y	Y	Y	Y	Y	Y	NA	Y	Y	Y	NA	Y	Y	Good
2018	Polak M, Colletti L, Colletti V^17^	Y	Y	Y	Y	Y	Y	Y	NA	Y	Y	Y	NA	Y	Y	Good
2018	Sung JKK, Luk BPK, Wong TKC, Thong JF, Wong HT, Tong MCF^12^	Y	Y	Y	Y	Y	Y	Y	NA	Y	Y	Y	NA	Y	Y	Good
2018	Asfour L, Friedmann DR, Shapiro WH, Roland Jr JT, Waltzman SB^8^	Y	Y	Y	Y	Y	Y	Y	NA	Y	Y	Y	NA	Y	Y	Good
2017	Wilkinson et al.^14^	Y	Y	Y	Y	Y	Y	Y	NA	Y	N	Y	NA	Y	Y	Good
2015	Yücel E, Aslan F, Özkan HB, Levent Sennaroğlu L^13^	Y	Y	Y	Y	Y	Y	Y	NA	Y	N	Y	NA	Y	Y	Good
2014	Bayazit YA, et al.^7^	Y	Y	Y	Y	Y	Y	Y	NA	Y	Y	Y	NA	Y	Y	Good

“Y”, Yes; “NA”, Not Applicable.

### Data extraction, synthesis and analysis

The following data were extracted from each included study: authors, year of publication, publication journal, place/country where the study was carried out, assessed age group, sample size, objective, results and conclusion.

A summary of the collected data containing authors, year of publication, place/country of the study, objectives, sample size, age of the assessed population and conclusions, was carried out in order to assess the benefits of the ABI in relation to aspects involving the development of auditory and language skills in the pediatric population (Table 2). Moreover, the data extracted from the studies were analyzed descriptively and comparatively.

**Table 2:** Information about the selected articles.

Authors/Year/Country	Objective	Sample N/age	Conclusion
Fernandes et al. (2020)/Brazil^4^	To characterize the development of hearing and language skills in children during the first 3 years of ABI use.	12 (2 yrs. to 11 yrs.)	Patients with ABI show slow and progressive development of hearing and language skills after activation.
Van der Straaten et al. (2019)/The Netherlands^15^	To assess long-term language development in children with prelingual deafness who received brainstem implants when compared to children who received cochlear implants (CIs) at the same hospital. Additional non-hearing impairments were taken into account.	10 (1.3 to 6.2 years)	For deaf children with bilateral inner ear malformations, the ABI provides satisfactory auditory input. Children with ABI are able to develop receptive and expressive language skills comparable to children with CIs with disabilities. Using this knowledge, preoperative counseling for parents can be refined.
Faes e Gillis (2019)/Belgium^3^	To investigate the development of spoken language after implantation. The lexical development of children with ABI is assessed longitudinally in comparison to children with typical hearing and children with CI.	12	Children with ABI develop spoken language skills. Their word usage steadily increases with longer ABI experience. Although there is still a difference in relation to children with CI and children of matched typical hearing age, the results are promising for the development of the spoken language of children with ABI.
Bayazit et al. (2014)/Turkey^7^	To provide information on the used methods and preliminary results for pediatric ABI (Auditory Brainstem Implant).	12 children	Auditory brainstem implants seem to be beneficial for some pediatric patients who cannot benefit from traditional cochlear implant surgery. The short-term benefits can be the recognition of ambient sounds, recognition of some commonly used words and phrases, and the start of word usage.
Eisenberg et al. (2018)/USA^9^	In the United States, the Food and Drug Administration authorized a Phase I clinical trial to determine the safety and feasibility of ABI.	10 children (2 to 5 years)	The ABI may be a viable option for children born with cochlear malformation and/or impaired auditory nerves, who do not show any apparent benefit from a CI. A multidisciplinary team is essential for performing the multiple assessments necessary to determine application and post-ABI follow-up. The ultimate benefits of this technology for most eligible pediatric candidates are not fully known at this stage. Still, the results of spoken communication cannot be predicted or normally expected, so the importance of maintaining continuous communication is a priority for these children, both pre- and post-ABI intervention.
Aslan et al. (2020)/Turkey^16^	To study the effect of age on the auditory brainstem implant (ABI) surgery on hearing perception, language and speech intelligibility.	30 children	ABI is a viable option to provide auditory sensations for children with cochlear abnormalities. ABI surgery under 3 years of age is associated with better hearing perception and language development when compared to older users.
Rajeswaran, Kameswaran (2020)/India^10^	To evaluate the safety and performance of auditory brainstem implant (ABI) communication in children with cochlear implant contraindications and without neurofibromatosis type II (NF2).	10 children (18 months to 18 years)	ABI provision and use are safe and allows a significant auditory development in children without NF2 who have contraindications for cochlear implants.
Baş, Yücel (2021)/Turkey^11^	To evaluate the relationship between phoneme recognition skills and language development skills in pediatric auditory brainstem implant (ABI) users. It also intends to identify delays and problems that may occur in the phoneme recognition skills of children with ABI and to shed light on rehabilitation programs.	20 children with ABI and 20 children with CI	Although children with ABI were not able to match the skills of their peers with CI, their language development and communication skills improved. It is believed that this study will contribute to the literature by demonstrating that the ABI use improves phoneme recognition skills in children who are not eligible for the CI or who do not adequately benefit from the CI.
Polak, Colletti and Colletti (2018)/Italy^17^	To develop a reliable and objective methodology for use with young children with auditory brainstem implants (ABI), through the ABR test. Moreover, to evaluate the intraoperative method using ABR to place the electrode in the brainstem, comparing the elicitability of the eABR test during ABI surgery and ABI processor activation.	17 young children implanted with ABI, with a mean age of 2 years and 4 months (8‒64 months).	The eABR test seems to be a reliable tool for assessing the ABI electrode placement and a reliable method for fitting children with an ABI. The data suggest that eABR-based fitting helps children to achieve hearing perception and development faster.
Sung et al. (2018)/China^12^	Retrospective review of the impact of ABI on audiological rehabilitation and language development of pediatric patients with profound prelingual deafness.	11 children with ABI (age group 1.67‒3.75 years)	Encouraging results in speech development were found, especially with the continuous use of the ABI. There was considerable variation in the results.
			Children with co-existing developmental cognitive and non-auditory disabilities did not perform as well. The Auditory Brainstem Implant is a safe and beneficial treatment for profound prelingual deafness in Cantonese-speaking pediatric patients.
Asfour et al. (2018)/USA^8^	To evaluate the results of auditory brainstem implant (ABI) in children in a prospective study.	12 children with ABI	The ABI is a good option for patients who are ineligible or fail the CI. Our results show that despite variable degrees of postoperative performance, the HRQoL ratings were positive. The presence of additional disabilities and health problems resulted in less positive HRQoL results. Our results emphasize the need to assess outcomes in these patients beyond speech perception and communication.
Wilkinson et al. (2017)/USA^14^	To determine the safety and feasibility of the Auditory Brainstem Implant (ABI) in children with congenital deafness with cochlear aplasia and/or cochlear nerve deficiency.	10 children, aged between 2 and 5 years	ABI surgery and device activation seem to be safe and feasible in this preliminary cohort.
Yücel, Aslan, Özkan, Levent Sennaroğlu (2015)/Turkey^13^	To describe the rehabilitation results in ABI users.	41 children with ABI	The subjects acquired basic audiological functions and were able to recognize and discriminate sounds in the third month of the ABI surgery.
			According to the time of ABI use and learning skills, the patients showed evolution from the identification of words to the level of sentence recognition within a wide spectrum.

## Results

### Selected studies

**Figure 1:**
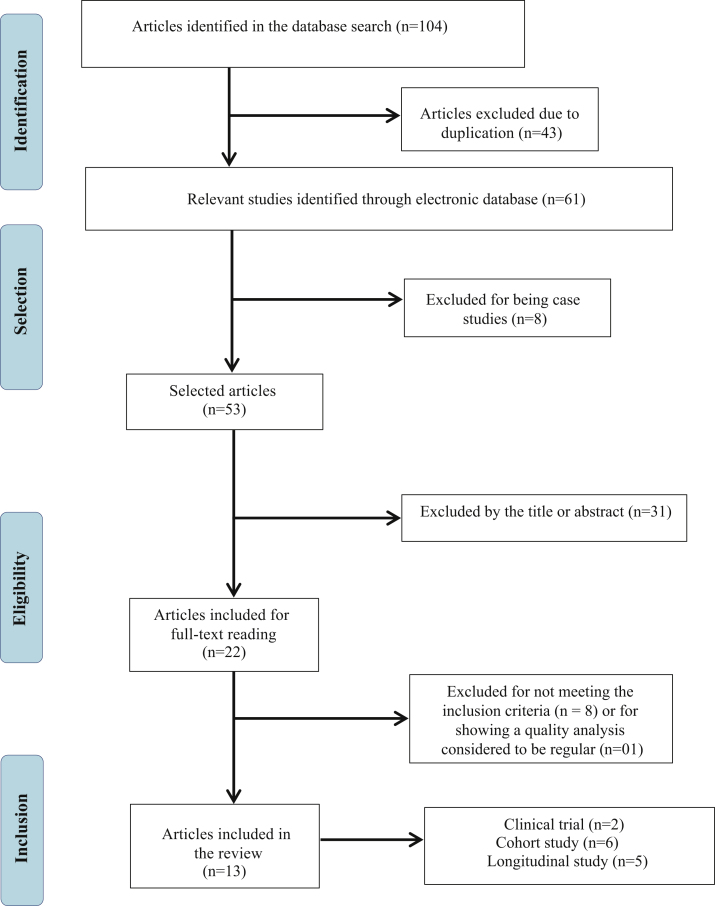
Flowchart of the article search and analysis process. Source: Prepared by the authors.

Initially, 104 publications were found, of which 43 were excluded because they were repeated articles and of the remaining 61, 8 were excluded because they were case studies. Thus, 53 publications had their titles and abstracts evaluated, 31 of which were excluded because they did not meet the eligibility criteria and, thus, 22 studies were selected to be read in full. Based on the reading of these articles, 08 were excluded for not meeting the eligibility criteria and, finally, after analyzing the quality of the publications, 01 was excluded because it was considered as having regular quality, that is, lower than that recommended to comprise the present review. Therefore, 13 articles were included in the present review study (Fig. 1), 03 of which focused on the auditory aspects, 03 with emphasis on the language development of children who underwent ABI and 07 publications contemplating both aspects.

### Characteristics of the selected studies

Based on the selected studies, it was observed that, although there is little research in the literature on the assessed topic, the ABI can be considered an effective alternative in child development.^7^, ^8^, ^9^, ^10^, ^11^

Regarding the benefits found in the studies that evaluated the auditory function, it can be observed that most children with ABI demonstrated detection of sounds in the postoperative period^12^ and were able to consistently respond to sounds, and to recognize, as well as discriminate sounds in the short term after the surgery.^7^, ^13^ Moreover, most children showed to be capable to detect the majority of speech sounds.^14^, ^15^ However, speech intelligibility was considered the most challenging skill.^16^ As for the methods used to assess the placement of the implanted electrodes, the eABR showed to be a good choice.^17^

Benefits were also found related to the aspects involving language development. Communication skills were present in children using ABI; however, the development was slow and progressive, and it is highlighted that visual communication is essential for post-implant intervention.^4^, ^9^ In the short term, most children identify sounds, respond to speech stimuli, and use their voice to attract attention.^15^ When compared to children with CIs, studies show that the population of children using ABI has poorer language development.^3^, ^11^, ^15^ Communication skills tend to show a significant increase in the short and long term in most cases; however, in some cases such skills remain stable.^10^ As for learning, children showed progress from word identification up to the sentence recognition level.^13^

## Discussion

### Benefit

The ABI has shown to be an alternative with significant benefits, regarding auditory and language skills, in cases of children with congenital cochlear malformation and/or auditory nerve impairment, who would not benefit from a CI surgery.^7^, ^8^, ^9^, ^10^, ^11^ It is important to note that when the ABI is implanted in children under three years of age, a better auditory perception and positive results in language development ensue.^16^

Although benefits have been observed regarding the use of the ABI, essentially to provide auditory sensations for children with cochlear anomalies,^16^, ^17^ the researchers point out that the development of communication^9^ and auditory skills^4^ is slow and progressive. Moreover, the need for a long-term intervention must be understood.^9^

When portraying children with other hearing-related impairments who require ABI, the development of auditory and language skills is even slower and more unpredictable, due to the few studies that assess these long-term factors and the range of associated factors, which can make research with larger populations difficult to perform.^8^, ^12^, ^13^

Therefore, the present research demonstrates that a careful selection with pre- and postoperative technical care, safety and benefit for the pediatric population after ABI surgery can be achieved.

### Language

Among the studies included in this review which deal with aspects of language, some quite recent report that children with ABI develop oral language after activating the device, but that occurs slowly and progressively.^4^, ^9^

As for the comparison of language aspects between children with ABI and children with CIs, some findings demonstrated that the language development is inferior in ABI users,^3^, ^11^, ^15^ and also that the ability to recognize two-syllable words and sentences is higher in CI than in ABI users.^18^

Rajeswaran and Kameswaran (2020)^10^ add that communication skills show a considerable increase from the preoperative period to 12 months, increasing significantly or remaining stable from 12 to 24 months, findings that are in agreement with those of another study,^19^ which also reviews long-term results and reports that it is possible to continue noticing improvements, even after one year of implant fitting.

As for speech intelligibility, after comparing groups of children using ABI with early and late intervention, recent research concluded that, in both groups, this was the most challenging skill regarding the development,^16^ as mentioned in almost all of the studies. Among these, there was also a report that the longer the ABI use, the greater the language evolution showed by the children, from the identification of words to the sentence recognition level.^13^

Children with associated disabilities, such as cognitive and/or additional health problems, had variable data among the findings in language and speech development, which could be expected due to the worsening of the general status of these children. As a result, there was a trend towards less positive responses that seem to delay the progression in these cases,^8^, ^12^, ^13^ when comorbidities are diagnosed.

Eisenberg et al. (2018) inferred in their study that visual support remains essential in the therapeutic intervention after ABI, since visual support expands the possibilities of learning the speech motor gesture and, that multiprofessional support and its different exchanges are necessary to better manage these cases, both in the selection and in the ABI fitting.^9^ These findings are in agreement with the results of a study^20^ that associates the fitting of the ABI to the use of orofacial reading as an important support for orality, and also points out the fundamental importance of a multidisciplinary approach in the favorable rehabilitation prognosis.

The use of scales and questionnaires aiming to measure the effects of ABI after surgery providing more information about the development of oral language, development of auditory stages, as well as quality of life, was mentioned in some studies.^4^, ^8^, ^15^

In a study carried out with 12 children aged between 2 and 11 years, it was observed that the maximum scores on the Infant Toddler Meaningful Auditory Integration Scale/Meaningful Auditory Integration Scale (IT-MAIS/ MAIS) and Meaningful Use of Speech Scale (MUSS), after 3 years of ABI use, were 45.35% and 32.28%.^4^ Another study with children using ABI, observed that 11 of 12 had some hearing benefit from their implant. It is noteworthy that parental assessments of Health-related Quality of Life (HRQoL) were positive for all domains, except communication.^8^

The protocols and scales are important in the monitoring of surgically implantable cases. The IT-MAIS, MAIS, MUSS scales have good reliability and validity, and can be used to measure effects on development and auditory and speech-language assessment, as in children with CIs.^21^

Among the mentioned articles, even though the majority showed that language development is progressive, variable responses were found and some of them emphasize that the continuous use of the ABI associated with prolonged intervention should be expected and encourage the continuous research beyond speech perception and communication in children using ABI.^8^, ^9^, ^12^

### Hearing

Regarding the assessment of the auditory aspects, most studies found that the ABI brings benefits to the pediatric population that cannot benefit from CI surgery. However, these studies highlight that, although benefits are observed in the development of children using ABI, benefits as satisfactory as in the cases of children using CI are not observed. In this sense, it is important to emphasize that the ABI provides levels of sound detection and discrimination that are similar to the CI, albeit without the tonotopic organization, which could justify the better results of the auditory aspects in the CI user population.^7^, ^9^, ^10^, ^22^^,^^23^

Children using ABI demonstrate sound detection in the postoperative period^12^; however, in this population, the development of auditory skills is slowly progressive.^4^, ^14^ While in some cases there is no constant response in relation to sounds, in others, the children are shown to respond consistently, and in the short term, they recognize environmental sounds, and some frequently used words and phrases.^7^ Moreover, researchers who evaluated 41 children using ABI observed that they all acquired basic auditory perception skills.^13^

Regarding speech detection thresholds, a study observed, in the sound field, thresholds around 30 to 35 dBHL with access to most speech sounds.^14^ Other authors^15^ evaluated the auditory performance of seven children with prelingual deafness who were followed for one year after the ABI; six of them identified sounds and reacted to speech. However, speech intelligibility was considered the most challenging skill,^16^ possibly because it has a higher level of complexity than pure tone and speech detection, requiring greater auditory performance.

In a study with children using Individual Sound Amplification Devices (ISAD), the level of open set/in silence speech discrimination ranged from 35% to 100% for both, ISAD and CIs.^22^

When using the IT-MAIS / MAIS questionnaire to assess the pediatric ABI users, researchers found a variation from 8 to 31 points (total of 40). These scores represent high variability and how much the process evolves as children begin to develop basic auditory skills.^14^ Moreover, children showed some capacity to discriminate words in a closed set.^14^ During the 12 months follow-up, children with ABI did not demonstrate spontaneous differentiation between two speakers or between speech sounds and non-speech sounds.^14^

In a study with patients with cochlear nerve impairment using bimodal stimulation, ABI and CI, the hearing thresholds with CI and ABI alone did not show a significant difference, and the auditory perception scores improved with the bimodal stimulation. The MAIS scores were significantly improved from unilateral CI to bimodal stimulation, and the pattern perception and word recognition were also significantly higher with the bimodal condition, when compared with the conditions of CI or ABI alone.^23^

The electrophysiological hearing tests in children with ABI showed evidence that the eABR is a good option to assess the placement of the implanted electrode and, thereby, to verify its position, which allows a better prognosis in auditory perception and language development.^17^

Based on the above information, the importance of carrying out more studies on the subject is highlighted, aiming at optimizing the intervention process and possible benefits in ABI users.

## Conclusion

Overall, the studies show that although the ABI has some surgical restrictions and contradictions in the literature, its use brings significant results for the global development of the pediatric population, even if they have a slower development of auditory and language skills when compared to children CI users.

The ABI can be considered an effective alternative for children with cochlear malformations and/or auditory nerve impairment, and those who cannot benefit from CI surgery. However, it is necessary to develop new studies to elucidate the best intervention approaches aiming to enhance the hearing and language development of these children.

## Conflicts of interest

The authors declare no conflicts of interest.
